# Supplementary material for: The mechanistic basis of cargo selection during Golgi maturation
Source: Sci Adv. 2025 Oct 3;11(40):eaea0016. doi: 10.1126/sciadv.aea0016 (PMC12494035; doi:10.1126/sciadv.aea0016)
Supplement: Supplementary file 1 — Figs. S1 to S7 Tables S1 and S2 References [file sciadv.aea0016_sm.pdf]

Science Advances
AAAS

Supplementary Materials for

The mechanistic basis of cargo selection during Golgi maturation

Rebecca J. Taylor *et al.*

Corresponding author: John A. G. Briggs, briggs@biochem.mpg.de; Sean Munro, sean@mrc-lmb.cam.ac.uk

Sci. Adv. **11**, eaea0016 (2025)
DOI: 10.1126/sciadv.aea0016

This PDF file includes:

Figs. S1 to S7
Tables S1 and S2
References

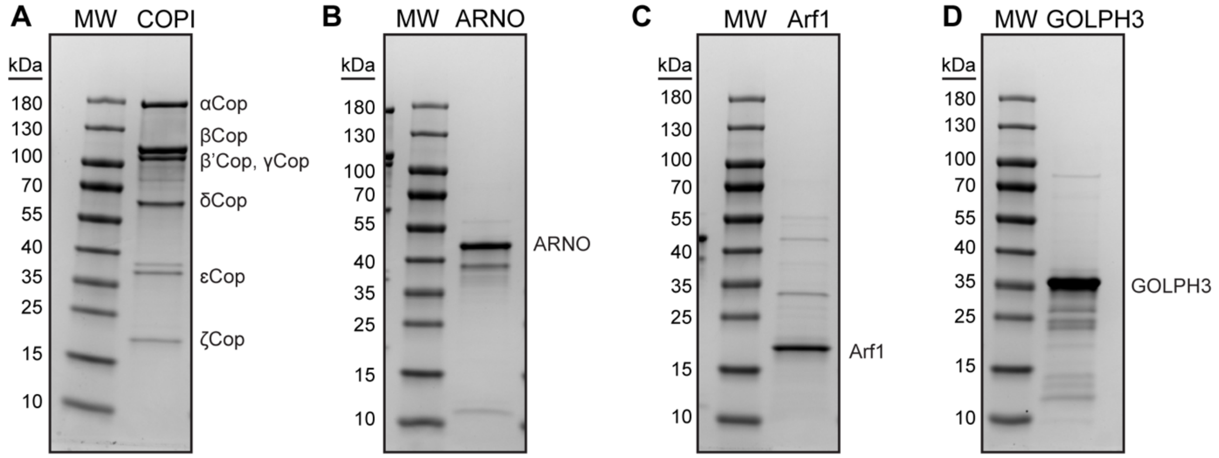

Fig. S1. Protein preparation for in vitro reconstitution of COPI-GOLPH3 coated vesicles from purified components.

(**A-D**) Coomassie gels for the protein components of the *in vitro* budding reaction: COPI (A), ARNO (B), Arf1 (C), and GOLPH3 (D).

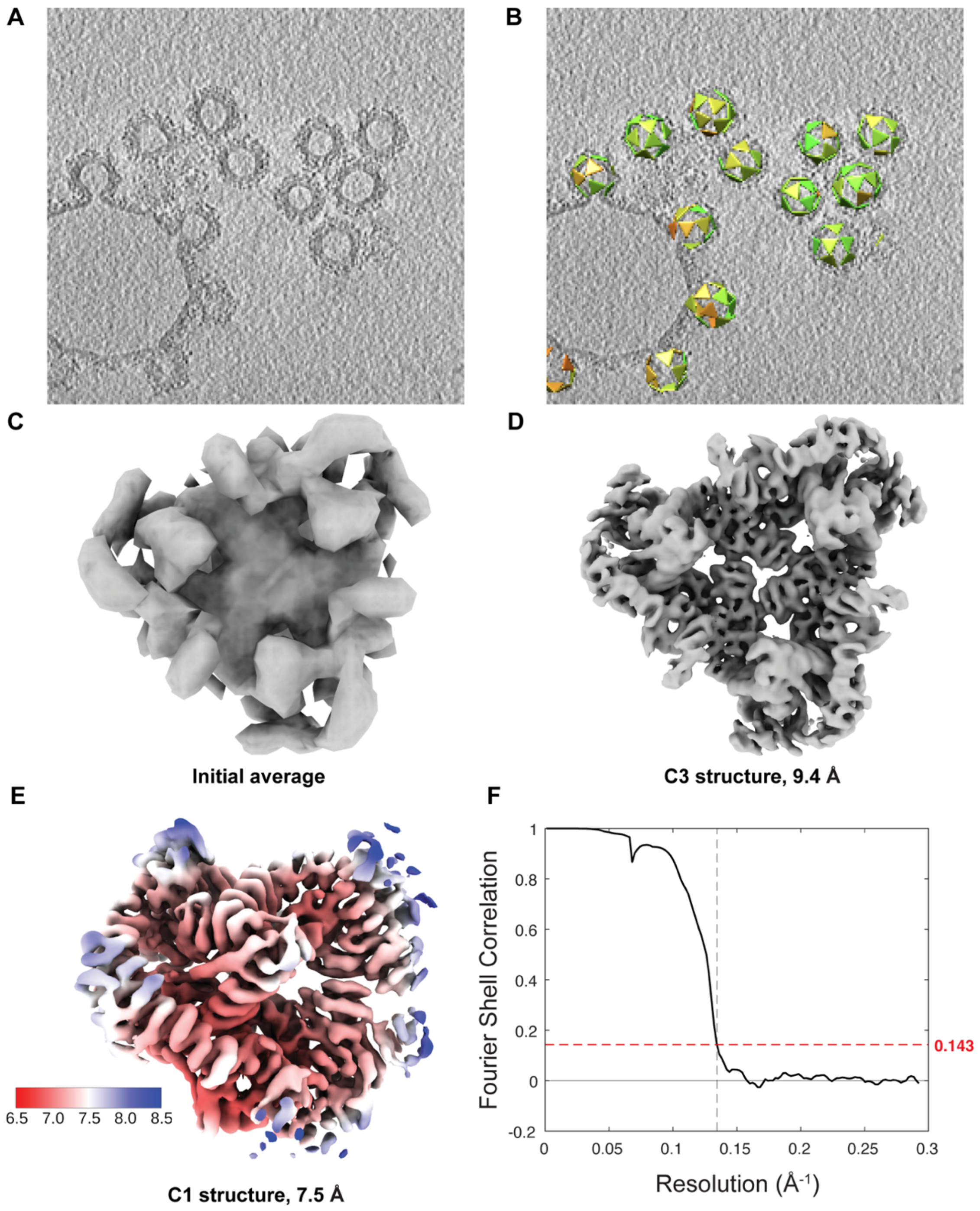

Fig. S2. Subtomogram averaging of COPI-GOLPH3 complex.

(**A**) Slice through a representative tomogram showing examples of COPI buds and vesicles.
(**B**) Aligned COPI triad positions marked by triangles, superimposed on the tomogram slice.
(**C**) initial average from SUBTOM picks after cleaning the particle list.
(**D**) C3-symmetric COPI-GOLPH3 structure at 9.4 Å resolution.
(**E**) C1 COPI-GOLPH3 structure obtained after symmetry expansion at 7.5 Å resolution.
(**F**) Fourier shell correlation plot. See also table S1.

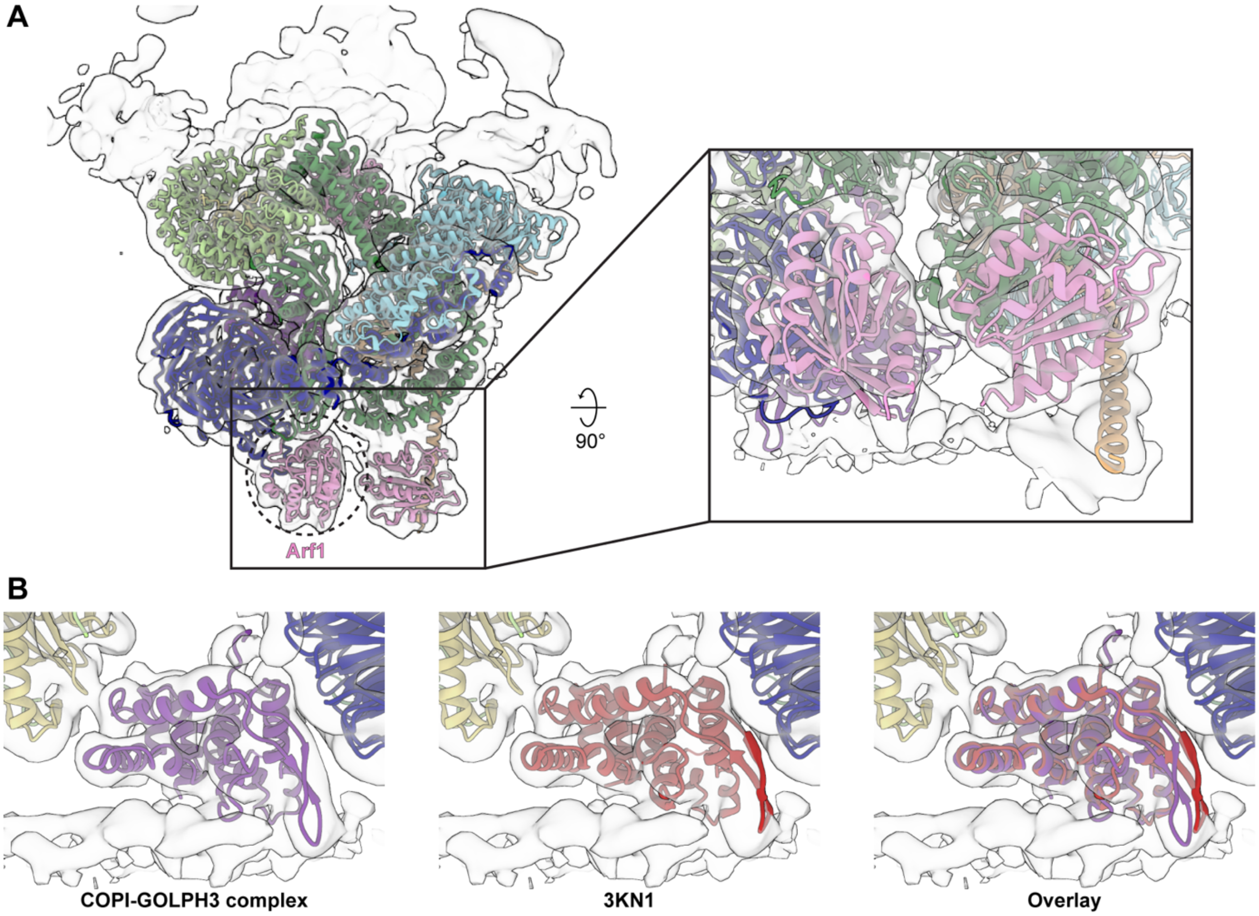

Fig. S3. Comparisons of COPI-GOLPH3 complex to COPI and GOLPH3 structures.

(**A**) The left panel shows the semi-transparent isosurface of the COPI leaf EM density map fitted with ribbon models of the COPI heptameric leaf containing GOLPH3 is shown from the vesicle exterior looking down toward the membrane. Dotted circle marks density adjacent to the N-terminal β-propeller of α-COP that was previously proposed to be constituted of a flexible loop in α-COP, but that can now be assigned as an additional copy of Arf1. The right panel shows a zoomed-in view, 90º-rotated to be perpendicular to the membrane to illustrate the fitting of the Arf1 ribbon model into the density. Components are coloured as follows: GOLPH3, purple, α-COP, dark blue; β-COP, dark green; β'-COP, light blue; δ-COP, orange; γ-COP, light green; ξ-COP, yellow, Arf1, pink.

(**B**) Semi-transparent isosurface fitted with a ribbon model of the COPI-GOLPH3 leaf. The view is centered on GOLPH3 and is perpendicular to the membrane**.** For comparison, the center panel, shows the density fitted with the crystal structure of GOLPH3 (PDB: 3KN1, (*21*)). The right panel shows an overlay.

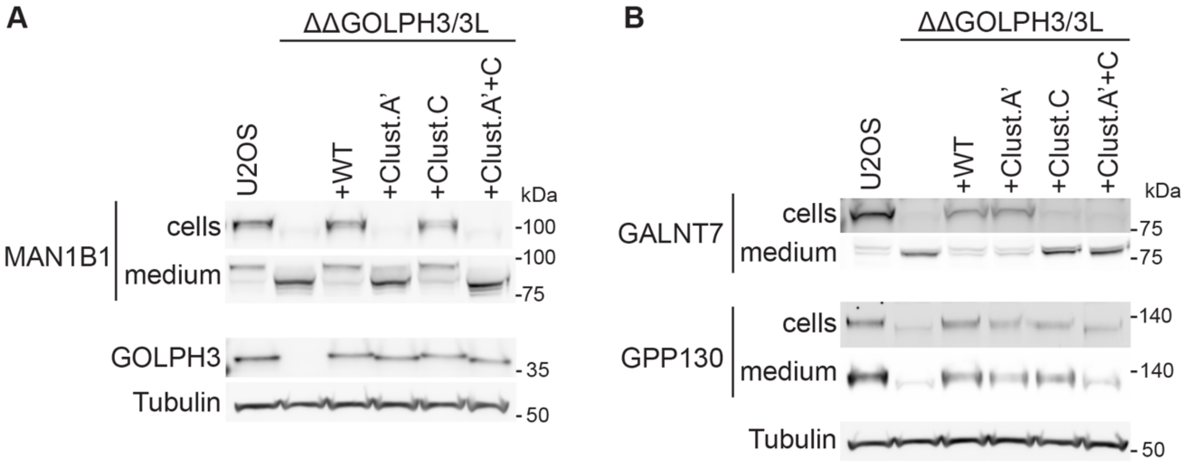

Fig. S4. Golgi residents destabilised by loss of GOLPH3 are either clipped and secreted, or degraded intracellularly.

(**A**) Immunoblots of whole-cell lysates and medium from either wild type U2OS cells or those lacking both GOLPH3 and GOLPH3L with the latter transfected with either wild-type GOLPH3 (WT) or the indicated mutant forms. Blots were labelled for the Golgi enzyme MAN1B1, GOLPH3 or tubulin as a loading control. When MAN1B1 is not retained by GOLPH3 it is released into the medium in a clipped form. Such clipping has been seen with other Golgi residents when their localisation is perturbed (*31*).

(**B**) Immunoblots of cells as in (A) but labelled for the Golgi residents GALNT7 and GPP130. When GALNT7 is not retained it is released into the medium in a clipped form. GPP130 does not appear in the medium at elevated levels when its cellular levels are reduced, consistent with previous reports that it is trafficked to the lysosome and degraded when not retained in the Golgi (*67*). We were unable to detect FAM3C in the medium and so could not determine its fate in the absence of GOLPH3-dependent retention.

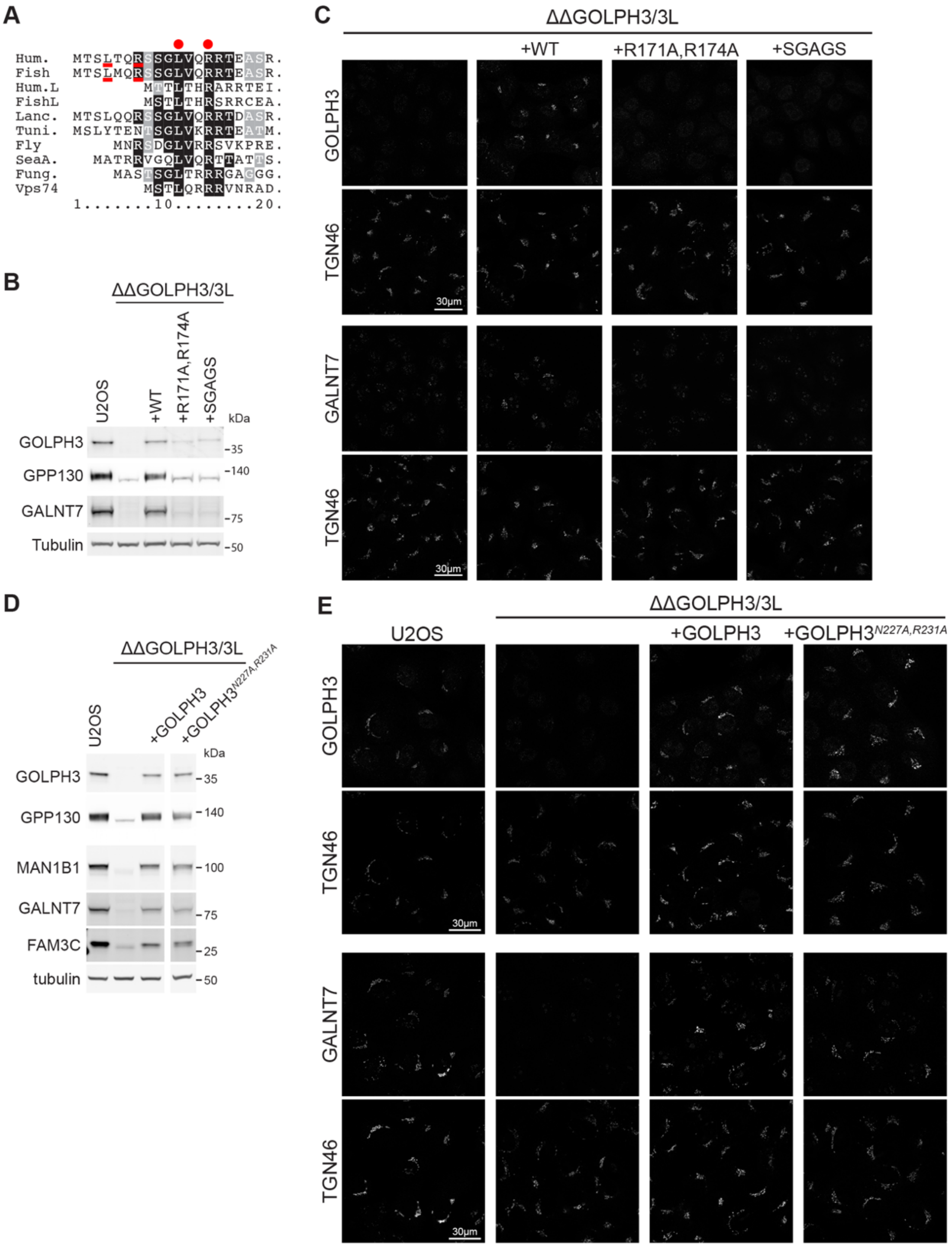

Fig. S5. Mapping of parts of GOLPH3 required for function.

(**A**) Alignment of the N-termini of GOLPH3 from the indicated species (Hum, human; Fish, coelacanth; Lanc, lancelet; Tuni, tunicate; Fly, *D. melanogaster*; SeaA, *Nematostella vectesis*; Fung, Aspergillus; Vps74, *S. cerevisiae*). Vertebrates also have GOLPH3L, as indicated. The conserved leucine and arginine at the ends of the region predicted to interact with coatomer are

indicated with red dots. In vertebrate GOLPH3, this motif appears to be present twice, as indicated with underlining.
(**B**) Immunoblot against GOLPH3 of whole cell lysates from U2OS cells deleted for GOLPH3 and GOLPH3L and expressing the indicated forms of GOLPH3. R171 and R174 are in the PI4P-binding site, and in SGAGS the hydrophobic hairpin residues $_{194}$FLLFD$_{198}$ are replaced with the eponymous sequence.
(**C**) Confocal micrographs of cells as in (B), labelled for the Golgi marker TGN46 and either GOLPH3, or the GOLPH3-dependent Golgi resident GALNT7.
(**D**) Immunoblots of whole cell lysates from U2OS ΔΔGOLPH3/3L expressing either nothing or GOLPH3 or GOLPH3 with mutations in residues near the possible interface with ζ-COP. Blots are labelled for GOLPH3 and the indicated Golgi residents or tubulin as a loading control, and the mutations do not appear to affect GOLPH3 function.
(**E**) Confocal micrographs of cells as in (D), labelled as in (C).

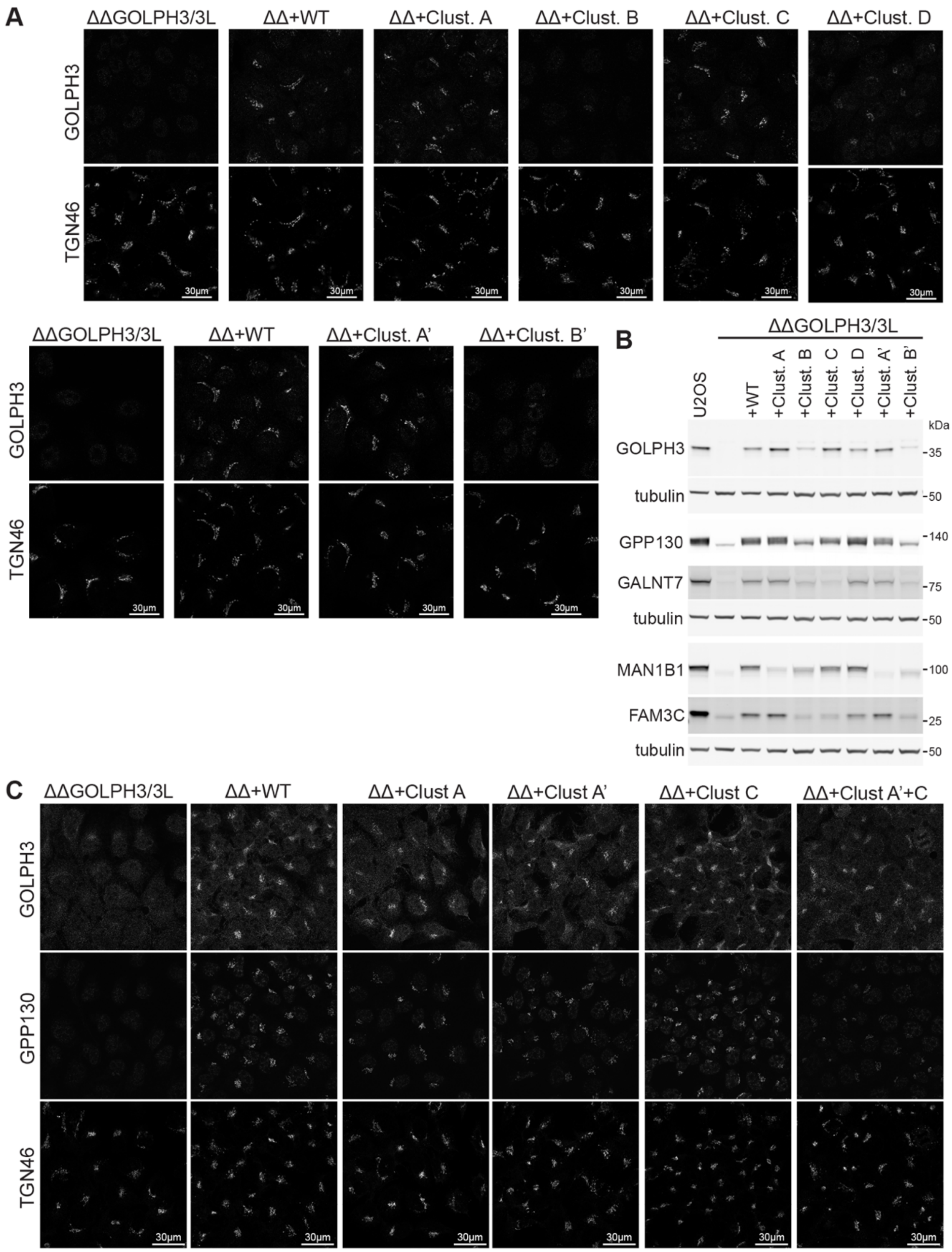

Fig. S6. Effect on GOLPH3 function of mutating clusters of acidic residues.

(**A**) Confocal micrographs of U2OS cells lacking GOLPH3 and GOLPH3L and transfected with either wild type GOLPH3 (WT), or forms with mutations to alanine of acidic residues in the indicated clusters. Cells were labelled for GOLPH3 and the Golgi marker TGN46.

(**B**) Immunoblots of whole cell lysates from U2OS cells lacking GOLPH3 and GOLPH3L and transfected with either wild type GOLPH3 (WT), or forms with mutations to alanine of acidic residues in the indicated clusters. Blots were labelled for GOLPH3 and the indicated Golgi residents or tubulin as a loading control. Mutations in different clusters affect the ability of GOLPH3 to rescue the stability of different residents.
(**C**) Confocal micrographs of U2OS cells lacking GOLPH3 and GOLPH3L and transfected with either wild type GOLPH3 (WT), or forms with mutations to alanine of acidic residues in the indicated clusters. Cells were labelled for GOLPH3, TGN46, and the GOLPH3-dependent Golgi resident GPP130.

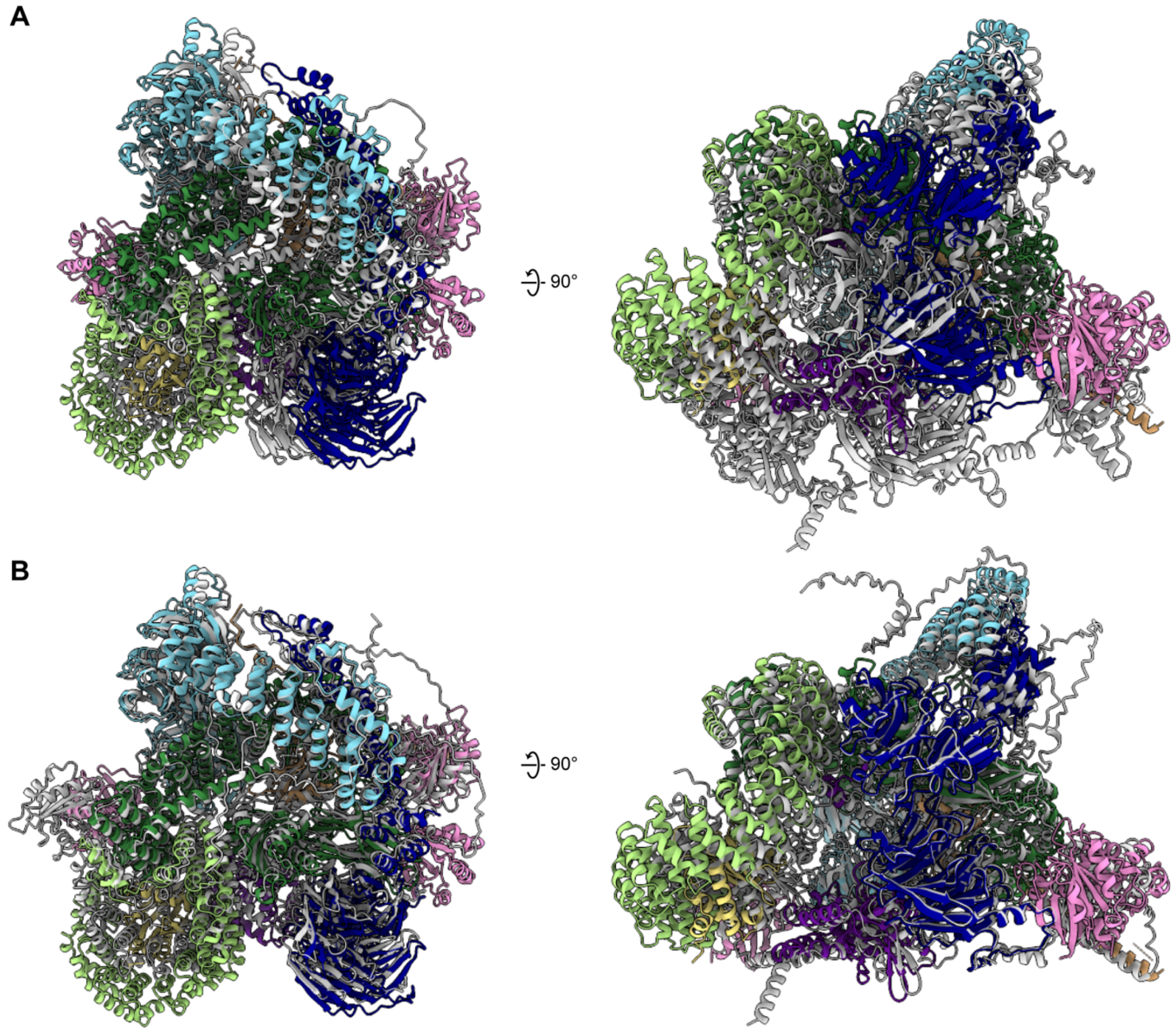

Fig. S7. Comparisons of Alphafold 3 predictions of the structure of GOLPH3 bound to COPI to the final model.

(**A**) The ribbon model of the COPI-GOLPH leaf (colored by component) aligned to a representative example of an Alphafold 3 prediction with the compressed conformation observed in approximately 70% of predictions (grey).
(**B**) The ribbon model of the COPI-GOLPH leaf (colored by component) aligned to a representative example of an Alphafold 3 prediction with a conformation similar to that observed in the experimental density, observed in approximately 30% of predictions (grey). A prediction with the conformation B was used as the starting point for model building.

Sample	COPI leaf with *M. musculus* COPI. *H. sapiens* Arf1, *H. sapiens* GOLPH3
**Data acquisition**	
Microscope	FEI Titan Krios
Voltage (keV)	300
Energy-filter (eV)	20
Detector	Gatan BioQuantum K3
Pixel size (Å)	1.701
Defocus range (microns)	-1.0 to -3.2
Acquisition scheme	-60/60˚, 3˚
Total Dose (electrons/Å^2)	125
Dose rate (electrons/Å^2/sec)	8.56
Frame number	10
Tomogram number	206
**Image processing**	
Vesicles and buds	4866
Subtomograms	108432
Symmetry	C1
Resolution at 0.143 FSC (Å)	7.5
B factor	-600

Table S1. CryoEM data acquisition and image processing.

Target	Host species	Source	Catalog number	RRID	Application	Working Dilution
**GOLPH3/3L**	Rabbit	Proteintech	19112-1-AP	AB_2113342	Western blot	1:1000
**GALNT7**	Rabbit	Abcam	ab97645	AB_10688428	Western blot	1:1000
**GALNT7**	Rabbit	Atlas Antibodies	HPA064243	AB_2685223	Western blot	1:1000
**GPP130**	Rabbit	Abcam	ab181849		Western blot (IMF)	1:1000 (1:200)
**MAN1B1**	Mouse	Merck	MABS1222		Western blot (IMF)	1:1000 (1:200)
**FAM3C**	Rabbit	Atlas Antibodies	HPA050548	AB_2681169	Western blot (IMF)	1:1000 (1:200)
**β-COP**	Rabbit	Abcam	ab2899	AB_2081300	Western blot	1:1000
**Tubulin**	Rat	John Kilmartin	YL1/2	AB_305328	Western blot	1:250
**GOLPH3**	Rabbit	Abcam	ab98023	AB_10860828	IMF	1:200
**GOLPH3**	Mouse	Proteintech	67777-1-IG	AB_2918542	IMF	1:200
**GALNT7**	Rabbit	Atlas Antibodies	HPA065317	AB_2685465	IMF	1:200
**TGN46**	Sheep	Bio-Rad	AHP-500G	AB_323104	IMF	1:200
**Rabbit Ig**	Donkey-AF680	Thermo Fisher	A32788	AB_2762831	Western blot	1:2000
**Mouse Ig**	Donkey-AF680	Thermo Fisher	A32802	AB_2762836	Western blot	1:2000
**Rat Ig**	Donkey-AF555	Thermo Fisher	A78945	AB_2910652	Western blot	1:2000
**Rabbit Ig**	Donkey-AF488	Thermo Fisher	A21206	AB_2535792	IMF	1:300
**Mouse Ig**	Donkey-AF488	Thermo Fisher	A21202	AB_141607	IMF	1:300
**Rabbit Ig**	Donkey-AF555	Thermo Fisher	A31572	AB_162543	IMF	1:300
**Mouse Ig**	Donkey-AF555	Thermo Fisher	A31570	AB_2536180	IMF	1:300
**Sheep Ig**	Donkey-AF555	Thermo Fisher	A21436	AB_2535857	IMF	1:300
**Rabbit Ig**	Donkey-AF647	Thermo Fisher	A31573	AB_2536183	IMF	1:300

Table S2. Primary and secondary antibodies used in this study.

Fluorescent secondary antibodies for immunofluorescence (IMF) and western blotting were labelled with Alexa Fluor (AF) dyes.

REFERENCES AND NOTES

1. B. S. Glick, A. Nakano, Membrane traffic within the Golgi apparatus. *Annu. Rev. Cell Dev. Biol.* **25**, 113–132 (2009).

2. M. G. Farquhar, G. E. Palade, The Golgi apparatus: 100 Years of progress and controversy. *Trends Cell Biol.* **8**, 2–10 (1998).

3. K. W. Moremen, M. Tiemeyer, A. V. Nairn, Vertebrate protein glycosylation: Diversity, synthesis and function. *Nat. Rev. Mol. Cell Biol.* **13**, 448–462 (2012).

4. K. T. Schjoldager, Y. Narimatsu, H. J. Joshi, H. Clausen, Global view of human protein glycosylation pathways and functions. *Nat. Rev. Mol. Cell Biol.* **21**, 729–749 (2020).

5. J. Béthune, F. T. Wieland, Assembly of COPI and COPII vesicular coat proteins on membranes. *Annu. Rev. Biophys.* **47**, 63–83 (2018).

6. N. Gomez-Navarro, E. Miller, Protein sorting at the ER-Golgi interface. *J. Cell Biol.* **215**, 769–778 (2016).

7. B. S. Glick, A. Luini, Models for Golgi traffic: A critical assessment. *Cold Spring Harb. Perspect. Biol.* **3**, a005215 (2011).

8. S. Emr, B. S. Glick, A. D. Linstedt, J. Lippincott-Schwartz, A. Luini, V. Malhotra, B. J. Marsh, A. Nakano, S. R. Pfeffer, C. Rabouille, J. E. Rothman, G. Warren, F. T. Wieland, Journeys through the Golgi—Taking stock in a new era. *J. Cell Biol.* **187**, 449–453 (2009).

9. Y. S. Bykov, M. Schaffer, S. O. Dodonova, S. Albert, J. M. Plitzko, W. Baumeister, B. D. Engel, J. A. Briggs, The structure of the COPI coat determined within the cell. *eLife* **6**, e32493 (2017).

10. R. J. Taylor, G. Tagiltsev, J. A. G. Briggs, The structure of COPI vesicles and regulation of vesicle turnover. *FEBS Lett.* **597**, 819–835 (2022).

11. E. C. Arakel, B. Schwappach, Formation of COPI-coated vesicles at a glance. *J. Cell Sci.* **131**, jcs209890 (2018).

12. L. P. Jackson, M. Lewis, H. M. Kent, M. A. Edeling, P. R. Evans, R. Duden, D. J. Owen, Molecular basis for recognition of dilysine trafficking motifs by COPI. *Dev. Cell* **23**, 1255–1262 (2012).

13. W. Ma, J. Goldberg, Rules for the recognition of dilysine retrieval motifs by coatomer. *EMBO J.* **32**, 926–937 (2013).

14. L. G. Welch, S. Munro, A tale of short tails, through thick and thin: Investigating the sorting mechanisms of Golgi enzymes. *FEBS Lett.* **593**, 2452–2465 (2019).

15. P. Lujan, F. Campelo, Should I stay or should I go? Golgi membrane spatial organization for protein sorting and retention. *Arch. Biochem. Biophys.* **707**, 108921 (2021).

16. L. Tu, W. C. S. Tai, L. Chen, D. K. Banfield, Signal-mediated dynamic retention of glycosyltransferases in the Golgi. *Science* **321**, 404–407 (2008).

17. K. R. Schmitz, J. Liu, S. Li, T. G. Setty, C. S. Wood, C. G. Burd, K. M. Ferguson, Golgi localization of glycosyltransferases requires a Vps74p oligomer. *Dev. Cell* **14**, 523–534 (2008).

18. E. S. P. Eckert, I. Reckmann, A. Hellwig, S. Röhling, A. El-Battari, F. T. Wieland, V. Popoff, Golgi phosphoprotein 3 triggers signal-mediated incorporation of glycosyltransferases into coatomer-coated (COPI) vesicles. *J. Biol. Chem.* **289**, 31319–31329 (2014).

19. L. G. Welch, S.-Y. Peak-Chew, F. Begum, T. J. Stevens, S. Munro, GOLPH3 and GOLPH3L are broad-spectrum COPI adaptors for sorting into intra-Golgi transport vesicles. *J. Cell Biol.* **220**, e202106115 (2021).

20. L. Tu, L. Chen, D. K. Banfield, A conserved N-terminal arginine-motif in GOLPH3-family proteins mediates binding to coatomer. *Traffic* **13**, 1496–1507 (2012).

21. C. S. Wood, K. R. Schmitz, N. J. Bessman, T. G. Setty, K. M. Ferguson, C. G. Burd, PtdIns4P recognition by Vps74/GOLPH3 links PtdIns 4-kinase signaling to retrograde Golgi trafficking. *J. Cell Biol.* **187**, 967–975 (2009).

22. S. Sechi, A. Frappaolo, A. Karimpour-Ghahnavieh, R. Piergentili, M. G. Giansanti, Oncogenic roles of GOLPH3 in the physiopathology of cancer. *Int. J. Mol. Sci.* **21**, 933 (2020).

23. S. S. Pinho, C. A. Reis, Glycosylation in cancer: Mechanisms and clinical implications. *Nat. Rev. Cancer* **15**, 540–555 (2015).

24. B. K. Brauer, Z. Chen, F. Beirow, J. Li, D. Meisinger, E. Capriotti, M. Schweizer, L. Wagner, J. Wienberg, L. Hobohm, L. Blume, W. Qiao, Y. Narimatsu, J. E. Carette, H. Clausen, D. Winter, T. Braulke, S. Jabs, M. Voss, GOLPH3 and GOLPH3L maintain Golgi localization of LYSET and a functional mannose 6-phosphate transport pathway. *EMBO J.* **43**, 6264–6290 (2024).

25. R. Rizzo, D. Russo, K. Kurokawa, P. Sahu, B. Lombardi, D. Supino, M. A. Zhukovsky, A. Vocat, P. Pothukuchi, V. Kunnathully, L. Capolupo, G. Boncompain, C. Vitagliano, F. Zito Marino, G. Aquino, D. Montariello, P. Henklein, L. Mandrich, G. Botti, H. Clausen, U. Mandel, T. Yamaji, K. Hanada, A. Budillon, F. Perez, S. Parashuraman, Y. A. Hannun, A. Nakano, D. Corda, G. D'Angelo, A. Luini, Golgi maturation-dependent glycoenzyme recycling controls glycosphingolipid biosynthesis and cell growth via GOLPH3. *EMBO J.* **40**, e107238 (2021).

26. H. J. F. Maccioni, R. Quiroga, W. Spessott, Organization of the synthesis of glycolipid oligosaccharides in the Golgi complex. *FEBS Lett.* **585**, 1691–1698 (2011).

27. S. O. Dodonova, P. Aderhold, J. Kopp, I. Ganeva, S. Röhling, W. J. H. Hagen, I. Sinning, F. Wieland, J. A. G. Briggs, 9Å structure of the COPI coat reveals that the Arf1 GTPase occupies two contrasting molecular environments. *eLife* **6**, e26691 (2017).

28. M. Faini, S. Prinz, R. Beck, M. Schorb, J. D. Riches, K. Bacia, B. Brügger, F. T. Wieland, J. A. G. Briggs, The structures of COPI-coated vesicles reveal alternate coatomer conformations and interactions. *Science* **336**, 1451–1454 (2012).

29. S. O. Dodonova, P. Diestelkoetter-Bachert, A. Von Appen, W. J. H. Hagen, R. Beck, M. Beck, F. Wieland, J. A. G. Briggs, A structure of the COPI coat and the role of coat proteins in membrane vesicle assembly. *Science* **349**, 195–198 (2015).

30. H. C. Dippold, M. M. Ng, S. E. Farber-Katz, S.-K. Lee, M. L. Kerr, M. C. Peterman, R. Sim, P. A. Wiharto, K. A. Galbraith, S. Madhavarapu, G. J. Fuchs, T. Meerloo, M. G. Farquhar, H. Zhou, S. J. Field, GOLPH3 bridges phosphatidylinositol-4- phosphate and actomyosin to stretch and shape the Golgi to promote budding. *Cell* **139**, 337–351 (2009).

31. M. Voss, Proteolytic cleavage of Golgi glycosyltransferases by SPPL3 and other proteases and its implications for cellular glycosylation. *Biochim. Biophys. Acta* **1868**, 130668 (2024).

32. Y. Cai, Y. Deng, F. Horenkamp, K. M. Reinisch, C. G. Burd, Sac1-Vps74 structure reveals a mechanism to terminate phosphoinositide signaling in the Golgi apparatus. *J. Cell Biol.* **206**, 485–491 (2014).

33. M. G. Farquhar, G. E. Palade, The Golgi apparatus (complex)-(1954-1981)-from artifact to center stage. *J. Cell Biol.* **91**, 77s–103s (1981).

34. W. E. Balch, W. G. Dunphy, W. A. Braell, J. E. Rothman, Reconstitution of the transport of protein between successive compartments of the golgi measured by the coupled incorporation of N-acetylglucosamine. *Cell* **39**, 405–416 (1984).

35. M. G. Waters, T. Serafini, J. E. Rothman, "Coatomer": A cytosolic protein complex containing subunits of non-clathrin-coated Golgi transport vesicles. *Nature* **349**, 248–251 (1991).

36. F. Letourneur, E. C. Gaynor, S. Hennecke, C. Démollière, R. Duden, S. D. Emr, H. Riezman, P. Cosson, Coatomer is essential for retrieval of dilysine-tagged proteins to the endoplasmic reticulum. *Cell* **79**, 1199–1207 (1994).

37. A. Pantazopoulou, B. S. Glick, A kinetic view of membrane traffic pathways can transcend the classical view of Golgi compartments. *Front. Cell Dev. Biol.* **7**, 153 (2019).

38. A. Nakano, A. Luini, Passage through the Golgi. *Curr. Opin. Cell Biol.* **22**, 471–478 (2010).

39. M. H. Dunlop, A. M. Ernst, L. K. Schroeder, D. K. Toomre, G. Lavieu, J. E. Rothman, Land-locked mammalian Golgi reveals cargo transport between stable cisternae. *Nat. Commun.* **8**, 432 (2017).

40. L. Orci, M. Ravazzola, A. Volchuk, T. Engel, M. Gmachl, M. Amherdt, A. Perrelet, T. H. Söllner, J. E. Rothman, Anterograde flow of cargo across the Golgi stack potentially mediated via bidirectional "percolating" COPI vesicles. *Proc. Natl. Acad. Sci. U.S.A.* **97**, 10400–10405 (2000).

41. T. Szul, E. Sztul, COPII and COPI traffic at the ER-Golgi interface. *Physiology* **26**, 348–364 (2011).

42. D. J. Stephens, N. Lin-Marq, A. Pagano, R. Pepperkok, J.-P. Paccaud, COPI-coated ER-to-Golgi transport complexes segregate from COPII in close proximity to ER exit sites. *J. Cell Sci.* **113**, 2177–2185 (2000).

43. T. Baba, A. Alvarez-Prats, Y. J. Kim, D. Abebe, S. Wilson, Z. Aldworth, M. A. Stopfer, J. Heuser, T. Balla, Myelination of peripheral nerves is controlled by PI4KB through regulation of Schwann cell Golgi function. *Proc. Natl. Acad. Sci. U.S.A.* **117**, 28102–28113 (2020).

44. Y. Liu, M. Boukhelifa, E. Tribble, E. Morin-Kensicki, A. Uetrecht, J. E. Bear, V. A. Bankaitis, The Sac1 phosphoinositide phosphatase regulates golgi membrane morphology and mitotic spindle organization in mammals. *MBoC* **19**, 3080–3096 (2008).

45. M. Ishida, ARMH3 is an ARL5 effector that promotes PI4KB-catalyzed PI4P synthesis at the trans-Golgi network. *Nat. Commun.* **15**, 10168 (2024).

46. M. Y. Hein, D. Peng, V. Todorova, F. McCarthy, K. Kim, C. Liu, L. Savy, C. Januel, R. Baltazar-Nunez, M. Sekhar, S. Vaid, S. Bax, M. Vangipuram, J. Burgess, L. Njoya, E. Wang, I. E. Ivanov, J. R. Byrum, S. Pradeep, C. G. Gonzalez, Y. Aniseia, J. S. Creery, A. H. McMorrow, S. Sunshine, S. Yeung-Levy, B. C. DeFelice, S. B. Mehta, D. N. Itzhak, J. E. Elias, M. D. Leonetti, Global organelle profiling reveals subcellular localization and remodeling at proteome scale. *Cell* **188**, 1137–1155.e20 (2025).

47. H. C. Tie, D. Mahajan, L. Lu, Visualizing intra-Golgi localization and transport by side-averaging Golgi ministacks. *J. Cell Biol.* **221**, e202109114 (2022).

48. M. C. Sahlmüller, J. R. P. M. Strating, R. Beck, P. Eckert, V. Popoff, M. Haag, A. Hellwig, I. Berger, B. Brügger, F. T. Wieland, Recombinant heptameric coatomer complexes: Novel tools to study isoform-specific functions. *Traffic* **12**, 682–692 (2011).

49. P. A. Randazzo, O. Weiss, R. A. Kahn, "[34] Preparation of recombinant ADP-ribosylation factor," in *Methods in Enzymology*, J. E. Rothman, Ed. (Academic Press, 1992), vol. 219 of *Reconstitution of Intracellular Transport*, pp. 362–369.

50. E. Mossessova, J. M. Gulbis, J. Goldberg, Structure of the guanine nucleotide exchange factor Sec7 domain of human arno and analysis of the interaction with ARF GTPase. *Cell* **92**, 415–423 (1998).

51. A. Spang, K. Matsuoka, S. Hamamoto, R. Schekman, L. Orci, Coatomer, Arf1p, and nucleotide are required to bud coat protein complex I-coated vesicles from large synthetic liposomes. *Proc. Natl. Acad. Sci. U.S.A.* **95**, 11199–11204 (1998).

52. W. J. H. Hagen, W. Wan, J. A. G. Briggs, Implementation of a cryo-electron tomography tilt-scheme optimized for high resolution subtomogram averaging. *J. Struct. Biol.* **197**, 191–198 (2017).

53. D. N. Mastronarde, Automated electron microscope tomography using robust prediction of specimen movements. *J. Struct. Biol.* **152**, 36–51 (2005).

54. J. R. Kremer, D. N. Mastronarde, J. R. McIntosh, Computer visualization of three-dimensional image data using IMOD. *J. Struct. Biol.* **116**, 71–76 (1996).

55. T. Grant, N. Grigorieff, Automatic estimation and correction of anisotropic magnification distortion in electron microscopes. *J. Struct. Biol.* **192**, 204–208 (2015).

56. Q. Xiong, M. K. Morphew, C. L. Schwartz, A. H. Hoenger, D. N. Mastronarde, CTF determination and correction for low dose tomographic tilt series. *J. Struct. Biol.* **168**, 378–387 (2009).

57. J. G. Galaz-Montoya, J. Flanagan, M. F. Schmid, S. J. Ludtke, Single particle tomography in EMAN2. *J. Struct. Biol.* **190**, 279–290 (2015).

58. B. Turoňová, F. K. M. Schur, W. Wan, J. A. G. Briggs, Efficient 3D-CTF correction for cryo-electron tomography using NovaCTF improves subtomogram averaging resolution to 3.4 Å. *J. Struct. Biol.* **199**, 187–195 (2017).

59. E. F. Pettersen, T. D. Goddard, C. C. Huang, G. S. Couch, D. M. Greenblatt, E. C. Meng, T. E. Ferrin, UCSF Chimera—A visualization system for exploratory research and analysis. *J. Comput. Chem.* **25**, 1605–1612 (2004).

60. K. Qu, Z. Ke, V. Zila, M. Anders-Össwein, B. Glass, F. Mücksch, R. Müller, C. Schultz, B. Müller, H.-G. Kraüsslich, J. A. G. Briggs, Maturation of the matrix and viral membrane of HIV-1. *Science* **373**, 700–704 (2021).

61. D. Tegunov, P. Cramer, Real-time cryo-electron microscopy data preprocessing with Warp. *Nat. Methods* **16**, 1146–1152 (2019).

62. J. Zivanov, T. Nakane, B. O. Forsberg, D. Kimanius, W. J. Hagen, E. Lindahl, S. H. Scheres, New tools for automated high-resolution cryo-EM structure determination in RELION-3. *eLife* **7**, 163 (2018).

63. D. Tegunov, L. Xue, C. Dienemann, P. Cramer, J. Mahamid, Multi-particle cryo-EM refinement with M visualizes ribosome-antibiotic complex at 3.5 Å in cells. *Nat. Methods* **18**, 186–193 (2021).

64. J. Abramson, J. Adler, J. Dunger, R. Evans, T. Green, A. Pritzel, O. Ronneberger, L. Willmore, A. J. Ballard, J. Bambrick, S. W. Bodenstein, D. A. Evans, C.-C. Hung, M. O'Neill, D. Reiman, K. Tunyasuvunakool, Z. Wu, A. Žemgulytė, E. Arvaniti, C. Beattie, O. Bertolli, A. Bridgland, A. Cherepanov, M. Congreve, A. I. Cowen-Rivers, A. Cowie, M. Figurnov, F. B. Fuchs, H. Gladman, R. Jain, Y. A. Khan, C. M. R. Low, K. Perlin, A. Potapenko, P. Savy, S. Singh, A. Stecula, A. Thillaisundaram, C. Tong, S. Yakneen, E. D. Zhong, M. Zielinski, A. Žídek, V.

Bapst, P. Kohli, M. Jaderberg, D. Hassabis, J. M. Jumper, Accurate structure prediction of biomolecular interactions with AlphaFold 3. *Nature* **630**, 493–500 (2024).

65. L. Liu, N. Watanabe, H. Akatsu, M. Nishimura, Neuronal expression of ILEI/FAM3C and its reduction in Alzheimer's disease. *Neuroscience* **330**, 236–246 (2016).

66. N. H. Cho, K. C. Cheveralls, A.-D. Brunner, K. Kim, A. C. Michaelis, P. Raghavan, H. Kobayashi, L. Savy, J. Y. Li, H. Canaj, J. Y. S. Kim, E. M. Stewart, C. Gnann, F. McCarthy, J. P. Cabrera, R. M. Brunetti, B. B. Chhun, G. Dingle, M. Y. Hein, B. Huang, S. B. Mehta, J. S. Weissman, R. Gómez-Sjöberg, D. N. Itzhak, L. A. Royer, M. Mann, M. D. Leonetti, OpenCell: Endogenous tagging for the cartography of human cellular organization. *Science* **375**, eabi6983 (2022).

67. S. Mukhopadhyay, C. Bachert, D. R. Smith, A. D. Linstedt, Manganese-induced trafficking and turnover of the *cis* -Golgi glycoprotein GPP130. *MBoC* **21**, 1282–1292 (2010).
